# Canalization of Language Structure From Environmental Constraints: A Computational Model of Word Learning From Multiple Cues

**DOI:** 10.1111/tops.12239

**Published:** 2016-12-18

**Authors:** Padraic Monaghan

**Affiliations:** ^1^ Department of Psychology Lancaster University; ^2^ Max Planck Institute for Psycholinguistics

**Keywords:** Canalization, Degeneracy, Language acquisition, Multiple cues, Word learning, Computational modeling

## Abstract

There is substantial variation in language experience, yet there is surprising similarity in the language structure acquired. Constraints on language structure may be external modulators that result in this canalization of language structure, or else they may derive from the broader, communicative environment in which language is acquired. In this paper, the latter perspective is tested for its adequacy in explaining robustness of language learning to environmental variation. A computational model of word learning from cross‐situational, multimodal information was constructed and tested. Key to the model's robustness was the presence of multiple, individually unreliable information sources to support learning. This “degeneracy” in the language system has a detrimental effect on learning, compared to a noise‐free environment, but has a critically important effect on acquisition of a canalized system that is resistant to environmental noise in communication.

## 1 Introduction

A key question in the cognitive sciences is how, despite the enormous variation in linguistic experience, the language learner acquires broadly the same language structure, “within a fairly narrow range” (Chomsky, 2005). This perspective has led to proposals for mechanisms that ensure canalization of language structure. Canalization refers to the means by which an individual's language is structured to support learning. Much of this structure may come from language itself, but there is growing realization that multiple, rich sources of information within the environment, rather than internally to the individual, may substantially constrain the language learning situation.

In biological evolution, canalization was once considered as a consequence of the natural selection of mechanisms that operate to minimize phenotypic variation (Waddington, 1942). For instance, Gallistel (1997) wrote of the default assumption in neuroscience that learning is a consequence of specialized mechanisms that are implemented as “organs within the brain.” Yet Wagner (1996) demonstrated that selecting for canalizing regulators required a rate of mutation that is higher than that observed in biological evolution, inconsistent with Gallistel's (2000) suggestion of modular, domain‐specific learning constraints within the individual.

To address this problem, an alternative perspective developed which proposed that minimal phenotypic variation, despite substantial environmental variation, is more likely to be stably achieved as a consequence of interaction between *multiple* regulators as part of the developmental process of the organism (Siegal & Bergman, 2002). In simulations of the operation of transcriptional regulators during development, they found that the greater the interactivity between these sources, the smaller the phenotypic variation resulting from environmental variation. Thus, canalization is a consequence of the process of development itself, realized through the effect of multiple interacting sources within the system, rather than additional moderators that apply to development.

An analogous perspective can be taken in canalization of social or cultural systems, such as language, whereby increasing levels of interaction may increase the stability and optimal processing of an information processing system (Bettencourt, 2009). Canalization, long conceived as being a consequence of mechanisms that implement resistance to environmental variation, can instead be the outcome of interacting, multiple sources of information.

Recently, there has been reconsideration of the potential richness of the language environment to support language learning. Instead of just focusing on the syntactic structure of utterances themselves, there have been recent moves to consider the multiple information sources supporting the situated language learner. For instance, supporting grammatical category acquisition, there is information from the distributional structure of language in terms of co‐occurrences of words (Redington, Chater, & Finch, 1998), but also substantial information from phonotactics and prosody distinguishing different grammatical categories, such as distinct stress patterns on nouns compared to verbs (Monaghan, Christiansen, & Chater, 2007). Furthermore, information about objects and actions within the child's purview may further constrain potential referents for words (Yurovsky, Smith, & Yu, 2013), providing constraining information about the semantic features associated with particular categories.

There have been several accounts for how multiple cues may be combined to support learning. The redundancy of different information sources may assist the child in increasing saliency of particularly important information in his or her environment (Bahrick, Lickliter, & Flom, 2004). Alternatively, the cues may operate summatively (Christiansen, Allen, & Seidenberg, 1998), or they may operate in a hierarchy, such that if one cue is available then it is used in preference to other cues, which are relied upon only if the preferred cues are unavailable (Mattys, White, & Melhorn, 2005).

An alternative perspective, consistent with views in biological evolution, is that the key function of multiple cues for language learning is their interactivity, resulting in a system stable to variation in the environment. This property of language is its “degeneracy,” defined as “the ability of elements that are structurally different to perform the same function or yield the same output” (Edelman & Gally, 2001). This degeneracy affects not only acquisition—where presence or absence of particular cues will not adversely affect the structure acquired—but also the robustness of the system once the language is acquired, due to reduced dependency on any one information source. Computational models of degeneracy in language and other complex systems have shown that it is important for robustness of learning (Whitacre, 2010), permitting, for instance, effective processing of speech sounds against background noise (Winter, 2014).

In this paper, a computational model of multiple interacting information sources is presented as a proof of concept of degeneracy resulting in canalization of language structure. The domain of study is word learning, where mappings have to be formed between words in utterances and the intended referent in the communicative environment. This task is difficult, due to numerous possibilities for the target candidate words in multi‐word utterances and equally numerous, even infinite, possible referents in the environment to which the target word may map (Quine, 1960). However, multiple cues are known to be available for assisting in constraining this task. These are present both in the spoken language and in the environment that surrounds the speaker and listener.

Within the spoken language itself, information about the grammatical roles for words can be ascertained from distributional information, consequently reducing the number of words in the utterance that need to be considered as the intended referring word. For instance, nouns are frequently preceded by articles (the, a) and these also tend to succeed verbs. Use of such simple distributional information has been shown to assist in determining word‐referent mappings (Monaghan & Mattock, 2012). Further information for identifying the critical information in an utterance is also available from prosodic information. When attempting to teach a child a new word, the speaker tends to increase the pitch variation, intensity, and duration of the target word within the utterance (Fernald, 1991). Thus, within‐language cues provide valuable multiple information sources to assist in word‐referent mappings.

In addition, constraints within the environment also help to reduce uncertainty about potential referents. One of these information cues is derived from cross‐situational statistical information. Even though, from a single learning trial, there may be several potential referents within the child's environment given an utterance, over multiple situations if, whenever a particular word is heard in speech, the target referent in the environment is also present, then the learner can increase his or her association between the target word and target object (McMurray, Horst, & Samuelson, 2012). Such cross‐situational learning (Yu & Smith, 2012) can further be supplemented by information that the speaker uses to indicate the field of reference. For instance, speakers tend to use deictic gestures (finger pointing or eye gaze) toward a referent which is being described (Iverson, Capirci, Longobardi, & Caselli, 1999).

However, each of these cues on its own is insufficient to perfectly constrain learning. The word succeeding an article is not always a noun—in English, adjectives might intervene, and spontaneous language is replete with false starts, and word sequencing errors. Similarly, the loudest word in speech is not always the target word, or a novel word being learned by the listener, and gestural cues are not always reliable. In Iverson et al.'s (1999) study they found that 15% of utterances were accompanied by gestures indicating aspects of the immediate environment to direct children's attention. However, such unreliability has profound value for learning. Consider if the child always learned from a speaker who reliably pointed to the intended referent. Then, if ever a situation arose where a referent was not gestured toward, this could impair effective communication, because the cue may be relied upon as part of the acquired word‐referent mapping.

There are costs to including multiple cues in the learning situation, because this increases the information required to be processed in each learning situation. So, the trade‐off between the increased strain on the cognitive systems required by processing of multiple as opposed to single, or no, cues and the potential advantages of interacting information sources for learning must be examined. In particular, the value of multiple information to support learning was tested, and also the importance of interaction among information sources in order to promote canalization—robustness of learning in the face of environmental variation.

A computational model was constructed to test integration of multiple sources of information to assist in learning relations between words and their referents. Two sets of simulations testing the model were conducted. The first assessed the contribution of single cues to word learning. The hypothesis was that adding cues to the input would assist in acquisition of the mapping—gestural cues assisting in defining the referent, prosodic cues promoting identification of the reference, and distributional cues supporting acquisition of both. However, the reliable presence of cues may also result in impaired ability to identify the form‐meaning mapping when the cue was no longer present.

The second set of simulations explored the role of multiple cues for learning. The prediction was that multiple cues would further promote learning, but that “degenerate” cues would be most effective for supporting not only effective acquisition but also robustness in the learning, immune from effects of variability in the environment. Thus, a model trained with a degenerate environment should be able to effectively map between words and referents even when environmental cues that support this mapping are no longer available.

## 2 A multimodal model of word learning

Previous models of integration of multiple information sources in computational models of language have been constructed in order to determine how informationally encapsulated each modality is in processing (e.g., Plaut, 2002). The starting point for the current model used the hub‐and‐spoke architecture, where information from different modalities is unconstrained in its integration. The model then determines the optimal way in which information sources can cohere to support learning. The model is closely based on a previous model of multimodal information integration in sentence processing to simulate behavior in the visual world paradigm (Smith, Monaghan, & Huettig, 2014, 2017). This modeling approach has a central processing resource that is connected to and from different sensory modalities, such as visual information about object identity, and auditory information about spoken word forms. This modeling framework has been effective in demonstrating how and when different information modalities interact in language processing, and how the influence of different modalities on language processing derives from the nature of the representations themselves, rather than requiring architectural assumptions to be imposed on the system.

The model used here is a simplification of this larger modeling enterprise, addressing the special case of acquiring word‐referent mappings. The model is compatible with previous associative models of word learning (McMurray et al., 2012), as well as being broadly consistent with the principles of statistical models of cross‐situational word learning (Yu & Smith, 2012). The model therefore applies these general modeling principles to explore the role of multiple information sources in facilitating, and constraining, word learning.

## 3 Architecture

**Figure 1:**
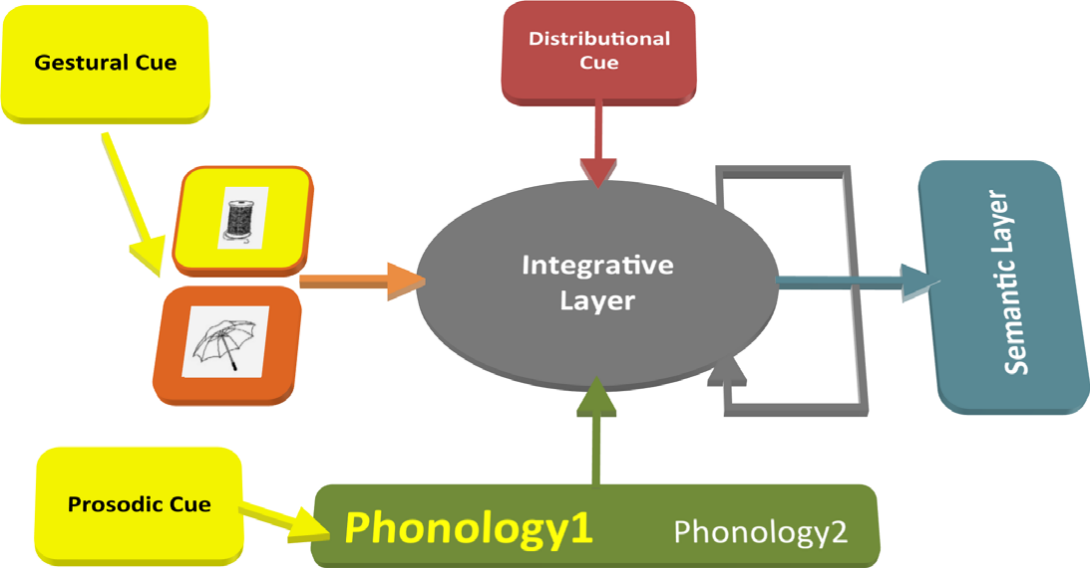
The multimodal integration model of word learning.

The model architecture is illustrated in Fig. 1. The model is implemented as a recurrent backpropagation neural network. It comprised a central hidden layer of 100 units which received connections from various input modalities and projected to a semantic layer output.

The phonological input represented two word slots, each of which contained 20 units. The visual input contained two locations each comprising 20 units, where object representations were presented. The semantic layer was composed of 100 units. For some simulations that included a distributional cue, the model also received input from a distributional cue layer, which was composed of 2 units. The integrative layer was also fully self‐connected.

## 4 Representations

The model was trained to learn 100 words. Representations of each modality of a word were encoded as a pseudopattern so that the properties of the relations between representations could be controlled. The phonological representation of each word was composed of four phonemes, randomly drawn from a set of 10 different phonemes. Each phoneme comprised 5 units, with 2 units active. The visual representation of the word's referent was constructed from 20 units with 8 units active for each representation. The semantic representations were localist, such that one of the 100 units was active for each of the words.

Fifty of the words were randomly assigned to one category, and the remaining 50 were assigned to the other category, such that these categories could be defined by a distributional cue.

## 5 Training

The model was trained to learn to identify the meaning of the word referred to by an input phonological and visual representation for all 100 words. Each trial was a simulation of a cross‐situational learning task, where two words and two objects were presented, but only one of the objects was named by one of the words (Monaghan & Mattock, 2012). The model had to learn to solve the task by generating the correct semantic representation for the named object.

For each training trial, a word was randomly selected. Its phonological form was presented at one of the two word slots in the phonological input (position was randomly chosen), and another randomly selected word's phonological form was presented at the other word slot. The object representation of the word's referent was presented at one of the two visual input positions (randomly chosen) and another randomly selected visual representation was presented at the other visual input position.

For the simulations with cues, gesture and prosody were implemented as intrinsic properties of the visual and phonological input representations, respectively, by doubling the activation at the input of the target visual object or the target phonological form. This had the effect of increasing the contribution of the target representation within each representational modality to affect the activation state of the integrative layer, and it was a simulation of increased saliency of that representation (i.e., that a gestural cue increases saliency of the target object, and prosodic cue is implemented as an increase in intensity, duration, and pitch of the target spoken word). This is illustrated in Fig. 1 as a highlighting of the uppermost object and the first phonological representation as a consequence of gestural and prosodic cues, respectively.

The distributional cue was implemented as an extrinsic cue. If the word was from the first (randomly assigned) category, then the first unit in the distributional layer was active, and if the word was from the second category, the second unit was active. This cue could therefore assist the model in determining which was the target object and spoken word, but the cue did not operate within either of these modalities.

The simulations of single cues presented each learning trial with the cue present with 100% reliability (see Table 1). The simulations of multiple cues varied the extent to which the cues were reliably present in each learning situation, from 0.25, through to 1.0 reliability.

**Table 1:** Proportion of training trials with each cue according to condition

Condition	Distributional Cue	Prosodic Cue	Gestural Cue
No cue	0	0	0
Single cues			
Dist cue	1	0	0
Prosodic cue	0	1	0
Gestural cue	0	0	1
Combined cues			
0.25 reliability	0.25	0.25	0.25
0.50 reliability	0.50	0.50	0.50
0.75 reliability	0.75	0.75	0.75
1.00 reliability	1	1	1

Activation cycled in the model for six time steps. At time step one, the visual and phonological inputs were presented. For two time steps, activation passed from the input to the integrative layer and from the integrative layer to the semantic layer, and from the integrative layer to itself. At time steps 3–6, the target semantic representation was presented at the semantic output layer, and activation continued to cycle around the model. The model was trained with continuous recurrent backpropagation through time (Pearlmutter, 1989) with error determined by sum squared error of the difference between the actual and target semantic representations. In one epoch of training, each of the 100 words occurred once as the target. The model was trained up to 100,000 epochs.

Twenty versions of the model with different pseudopattern representations, different randomized starting weights, and different randomized ordering of training patterns were run.

## 6 Testing

The model's performance was assessed during training on its ability to produce the target semantic representation for each phonological and visual input. If the activity of the semantic unit corresponding to the target word was more active than any of the other units in the semantic layer, then the model was determined to be accurate.

Accuracy during training was assessed, and also the number of epochs at which the model was able to accurately detect all 100 words for five consecutive epochs.

At the end of training, the robustness of the model's learning was assessed by measuring its accuracy when no cues were present during testing.

## 7 Results

### 7.1 Single cues

**Figure 2:**
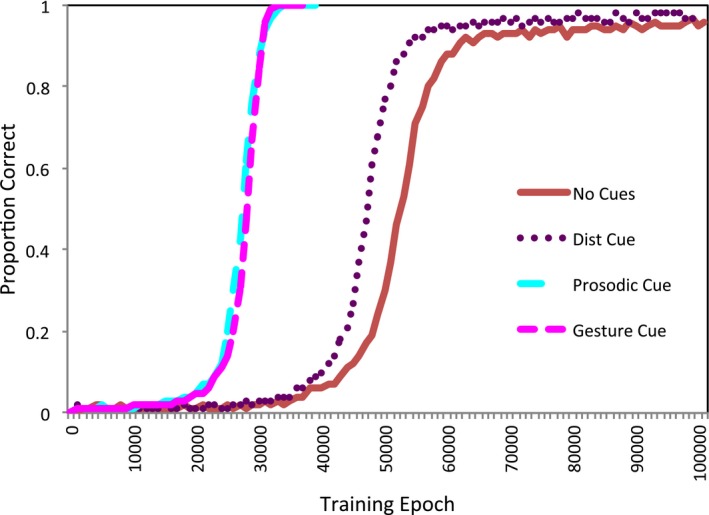
Accuracy during training for the single cues conditions, compared to the no cue condition.

The model's accuracy during training when no cues or single cues were present is shown in Fig. 2.

An anova with time taken to reach criterion as dependent variable, and cue condition (no cue, distributional cue, prosodic cue, gestural cue) as within subjects factor, was conducted to test whether the model learned differently according to the presence of cues. The result was significant, *F*(3, 57) = 70,722, *p* < .001, η_p_^2^ = 1.00. Post hoc tests revealed that the model learned to criterion more quickly for the prosodic cue (mean epochs = 35,800, *SD* = 1,005) and gestural cue (*M* = 35,650, *SD* = 745) conditions than the no cue condition, which had not reached criterion by 100,000 epochs (mean proportion correct was 0.96), both *p* < .001. Though the trajectory of learning was distinct, as shown in Fig. 2, the effect of distributional cues was smaller, and not significantly different in time to criterion compared to the no cue condition (mean proportion correct after 100,000 epochs was 0.99). The prosodic and gestural cues supported learning more than the distributional cue, both *p* < .001, but there was no statistical difference in speed of learning from the prosodic and gestural cues, *p* = 1.

**Figure 3:**
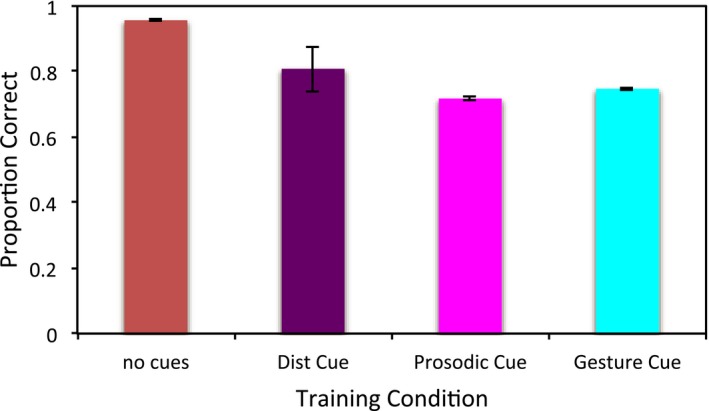
Accuracy after training for the single cues conditions, when no cues are present during testing.

The robustness of the model's learning to omission of cues during testing is shown in Fig. 3. An anova on accuracy in the post‐learning test with no cues present, and cue condition as within subjects factor was significant, *F*(3, 57) = 8.982, *p* < .001, η_p_^2^ = 0.321. Post hoc tests showed that the distributional cue did not significantly affect robustness of learning compared to the no cue condition, *p* = .284; however, the prosodic and gestural cue both resulted in poorer performance than the no cue condition, both *p* < .001. The gestural cue resulted in more robust learning than the prosodic cue, *p* = .001, but these conditions did not differ significantly from the distributional cue condition, both *p* = 1.

Next, the difference between the intrinsic cue conditions (prosodic and gestural cues) was assessed to determine if this was due to their quicker acquisition. Every model was trained to the same number of training trials (100,000), then tested for robustness of learning. The results were similar. Even with more training, the effect of a single, reliable intrinsic cue was detrimental to the model's ability to map between form and meaning when the cue was not present, *F*(3, 48) = 45.62, *p* < .001, η_p_^2^ = 0.740. Prosodic and gestural cues were now not significantly different than one another, *p* = .423, but were both significantly different than no cue and the distributional cue conditions, all *p* < .001.

### 7.2 Multiple cues

**Figure 4:**
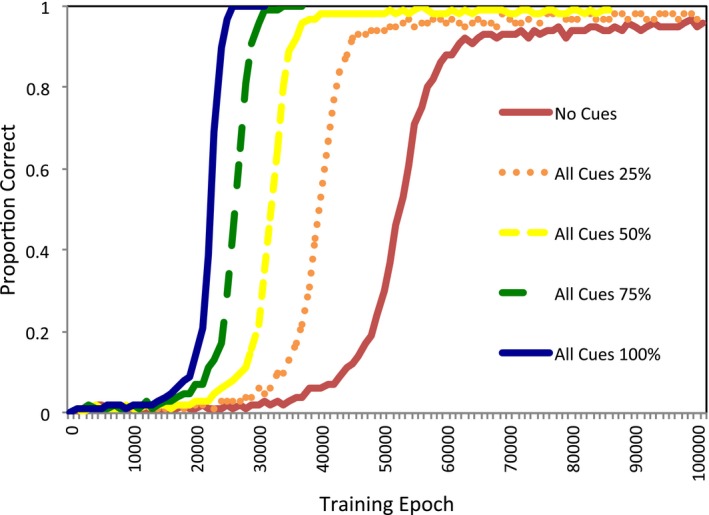
Accuracy during training for the multiple cue conditions, compared to the no cue condition.

The model's accuracy during training for combined cues with different levels of reliability is shown in Fig. 4. For time taken to reach training criterion, an anova indicated that combined cues with different reliability resulted in a significant effect on speed of learning, *F*(4, 76) = 3,855, *p* < .001, η_p_^2^ = 0.99. Post hoc tests indicated that the no cue and the 0.25 cue reliability conditions were significantly slower in learning than the 0.50 condition, both *p* < .001, which was in turn slower than the 0.75 condition, *p* < .001, which was in turn slower than the 1.00 perfect reliability multiple cue condition, *p* < .001. Thus, as anticipated, the greater the reliability of information, the faster the model learned to map between forms and meanings.

**Figure 5:**
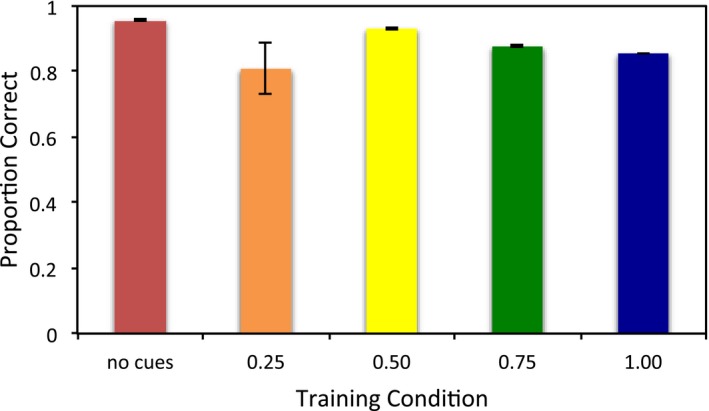
Accuracy after training for the multiple cue conditions, when no cues are present during testing.

The robustness of learning was also compared between these conditions. The results are shown in Fig. 5. An anova demonstrated that the robustness of performance at testing was affected by the cues presented during training, *F*(4, 76) = 2.953, *p* = .025, η_p_^2^ = 0.135. Post hoc tests revealed that the no cue and 0.50, 0.75, and 1.00 cue conditions were significantly different, all *p* < .001. The 0.25 cue condition was not significantly different than any other condition, all *p* ≥ .718. As reliability increased from 0.50 to 0.75, the robustness of the model declined, *p* < .001, and similarly declined from 0.75 to 1.00 reliability, *p* < .001. Thus, low reliability of cues did not seem to assist in learning quickly or robustly, but once individual cues appeared at least half the time, increasing further the reliability of the cues began to reduce the resistance of the model to the absence of cues after training.

## 8 Discussion

Language learning occurs in situations where multiple, interacting sources of information are available to support the learning. However, attending to multiple cues increases the processing load on the individual. However, this degeneracy in language results in two important advantages for the language learning system.

First, adding a combination of cues improves the speed and accuracy of learning to map between representations. Providing some guiding information about the intended object in a scene containing more than one referent and information about the intended reference in an utterance containing more than one word, along with additional information about the general category of the target, improves performance. Even when the individual cues occur only 50% of the time, this still resulted in a significant advantage for acquisition of form‐meaning mappings compared to no cues being present at all.

The second advantage of the degeneracy of language is that the learning that is acquired from a degenerate environment is highly robust (Ay, Flack, & Krakauer, 2007), and the model was able to make use of cues even when they were variable in their presence across communicative situations. However, this multiple cue advantage for robustness was only observed when there was noise in the environment: When the cues occurred with perfect reliability then, even though learning was optimal in speed, the acquired system was brittle and prone to error under suboptimal subsequent conditions. Thus, canalization of language structure can be conceived of as a consequence of the interaction of multiple information sources for learning. There is therefore a trade‐off between speed of initial learning and the robustness of that learning. The former is supported by perfectly reliable information, and more information resulted in better learning. The latter is supported by multiple information sources, but with each individual source being somewhat noisy. The precise point of this trade‐off is an issue for further exploration in computational systems, to determine the extent to which natural language environments are optimally designed for acquisition.

Chomsky (2005) wrote of the problem of canalization as a “fair description of the growth of language in the individual,” in that “a core problem of the faculty of language is to discover the mechanisms that limit outcomes to ‘optimal types,’” referring to the constraints of syntax. The current simulations demonstrate for word learning that these constraints may not be language‐specific mechanisms within the learner, but rather the response of a general‐purpose learning system that produces constraints as a consequence of integration of multiple cues from the environment. But do these principles of word learning apply also to acquisition of syntax, which has largely been the domain in which the problem of canalization has been discussed (e.g., Chomsky, 2005; Newmeyer, 2004)?

The observation that speed and accuracy of word learning are promoted by multiple cues is consistent with several current accounts of multiple cue integration for various language learning tasks (see Monaghan & Rowland, in press; for a review). For instance, for speech segmentation, Monaghan, White, and Merkx (2013) assessed the acoustic properties of speech to identify multiple prosodic cues that can combine to promote identification of words. In the same domain, Christiansen et al. (1998) provided a computational model that demonstrated how multiple cues boost segmentation. Relatedly, Mattys et al. (2005) presented a theoretical model for how multiple cues from speech may cohere to promote speech recognition. Similarly, for learning word‐referent mappings, the current multiple cues model is consistent with Bahrick et al.'s (2004) model of intersensory redundancy for word learning, where multiple cues are vital for guiding the child toward informative properties of the environment.

These language learning tasks all concern identification of information that could potentially be processed using associative learning mechanisms (Yu & Smith, 2012); so the question remains whether there is evidence that language structure in terms of morphology and syntax could also be constrained by multiple cues. Again, there is converging evidence for these language learning tasks that multiple cues play a key role—not only co‐occurrence constraints between morphemes or between words (Fries, 1952), but also phonological and prosodic properties of words can constrain identification of grammatical categories of words (Kelly, 1992; Monaghan et al., 2007) and facilitate learning of non‐adjacent dependencies (Newport & Aslin, 2004). It is of course possible that a completely different process applies for syntax acquisition than learning all other aspects of language, but another starting point to acquisition is that such constraints emerge from the same general statistical learning mechanisms: Learning of structure of words, grammatical categories, and syntax are not distinct processes (Frost & Monaghan, 2016).

Nevertheless, a common feature of all these multiple cue studies of language learning is that they would predict the growing advantage of learning as cues increase in reliability, as observed in the current simulations. The simulations presented here suggest that, rather than canalization being a challenge in the face of environmental variation, it is instead a primary consequence of this variation in a system that is able to integrate multiple information sources.
